# Research progress on traditional Chinese medicine-assisted immune checkpoint inhibitors therapy for solid tumors

**DOI:** 10.3389/fimmu.2026.1723860

**Published:** 2026-01-30

**Authors:** Zhipeng Liu, Yijie Xie, Yi He, Keyu Chen, Minjie Zhou, Guodong Ha, Lincheng Duan, Zhengyu Zhao, Dingjun Cai

**Affiliations:** Acupuncture and Tuina School, Chengdu University of Traditional Chinese Medicine, Chengdu, China

**Keywords:** drug delivery, immune cell infiltration, immune checkpoint inhibitors, solid tumors, traditional Chinese medicine, tumor microenvironment

## Abstract

Immune checkpoint inhibitors (ICIs) represent a promising approach in tumor therapy. However, when applied to solid tumors—the predominant form of tumors—their efficacy is often constrained by the distinctive tumor microenvironment, yielding low response rates and resistance. Additionally, treatment with ICIs commonly leads to immune-related adverse events (irAEs). These challenges have restricted the broader clinical application of ICIs, underscoring the need for strategies that can enhance their antitumor effects while maintaining a favorable safety profile. Traditional Chinese medicine (TCM) is characterized by holistic regulation and has the therapeutic advantages of multi-level, multi-target and low toxicity. In recent years, it has shown great potential in addressing ICIs-related limitations. This review systematically summarizes current advances in the combined use of TCM and ICIs for solid tumors, aiming to offer new perspectives for incorporating TCM into future cancer treatment strategies.

## 1 Introduction

The worldwide burden of cancer keeps on increasing. Around 20 million new cancer cases and 9.7 million deaths caused by cancer were recorded worldwide in 2022 (1), representing a 41.8% increase in new cases and an 18.3% rise in deaths compared to 2012 (2). Notably, solid tumors constitute the majority of this burden. In 2022, nine of the top ten cancers with the highest mortality were solid tumors, with lung, colorectal, and liver cancers alone accounting for 18.7%, 9.3%, and 7.8% of total cancer deaths, respectively (1). This concerning trend emphasizes the urgent requirement for more effective treatment strategies.

In recent years, advances in the study of the TME and tumor immunology have established ICIs as a major therapeutic approach in tumor treatment (3). Together with surgery, chemotherapy, and radiotherapy, ICIs now represent a cornerstone of tumor treatment. However, the unique characteristics of the TME in solid tumors impose significant limitations on the clinical efficacy of ICIs (4). For instance, in non-small cell lung cancer (NSCLC)—the most common solid tumor—though immune checkpoint inhibitors (ICIs) have achieved breakthroughs in tumor treatment, only 20%-30% of patients respond to ICIs therapy (5). And a phase II clinical trial investigating nivolumab combined with ipilimumab for NSCLC demonstrated that 80% of patients experienced treatment-related adverse events, while 29% developed grade 3 or 4 treatment-related adverse events (6). Moreover, a meta-analysis indicates that the objective response rate (ORR) after ICIs rechallenge (19.4%) was significantly lower than the pooled ORR across the entire treatment period (31.4%), with a pooled odds ratio of 0.36 (*P* < 0.00001). Additionally, the disease control rate after rechallenge decreased from 59.2% to 54.8%, with a pooled odds ratio of 0.62 (*P* = 0.010) (7). Enhancing the efficacy of ICIs while minimizing associated adverse events remains a critical challenge in clinical practice.

TCM has consistently shown distinct benefits in cancer treatment. As early as the period of the *Yellow Emperor’s Inner Canon*, TCM had already documented understanding of tumors. For instance, the *Ling Shu·Ci Jie Zhen Xie Lun* describes the etiology and pathogenesis of various tumors such as tendon tumor, chronic tumor, intestinal tumor, bone tumor, and fleshy tumor, providing a theoretical foundation for later generations in treating tumor-like diseases. Through syndrome differentiation and treatment, TCM can develop personalized therapeutic strategies tailored to individual patient differences, thereby optimizing treatment outcomes. Furthermore, both the special processing methods and drug compatibility of TCM can reduce the toxic and side effects of drugs (8, 9). There were also studies that evaluated and controlled the safety of traditional Chinese medicine based on ADME (Absorption, Distribution, Metabolism, Excretion) data (10, 11). Therefore, TCM may serve as an effective approach to improve the efficacy of ICIs and mitigate irAEs. Over the past few years, an increasing number of studies have shown that TCM holds considerable potential when used in conjunction with immunotherapeutic approaches (12). Against this backdrop, this review analyzes and summarizes existing literature to explore the research progress on TCM-assisted ICIs therapy for solid tumors. It aims to provide new insights for clinical tumor therapy, promote the broader application of TCM in tumor immunotherapy, and contribute to the development of integrated TCM and Modern medicine anti-tumor system.

## 2 Research progress in ICIs

ICIs are monoclonal antibodies that primarily fall into three classes in clinical use. They specifically target cytotoxic T lymphocyte-associated antigen-4 (CTLA-4), programmed cell death protein-1 (PD-1), and programmed cell death ligand-1 (PD-L1), respectively. By interfering with the function of these immune checkpoints, ICIs alleviate tumor-induced immunosuppression, potentiate antitumor immunity, and ultimately inhibit tumor growth.

### 2.1 Mechanisms and functions of ICIs

CTLA-4, a suppressive receptor belonging to the CD28 family, shares a pair of ligands—CD80 and CD86—with CD28. When CD28 binds to CD80 or CD86, it generates a co-stimulatory signal. This signal not only reduces the effective threshold needed for T cell activation (13) but also amplifies the activation signal triggered by the interaction between the T cell receptor (TCR) and the MHC complex on antigen-presenting cells (14), with these combined effects driving T cell activation and proliferation. Conversely, CTLA-4 exerts an inhibitory effect. On resting T cells, CTLA-4 shows low-level expression on the cell surface, and it is primarily distributed in the Golgi apparatus and lysosomes. Only after T cells are activated by TCR signals and CD28 costimulatory signals is CTLA-4 translocated to the T cell surface. It then exerts an inhibitory effect on T cell activity via the synergy of intrinsic and extrinsic regulatory pathways: intrinsically, it suppresses proximal TCR and CD28 signaling and competitively binds to the two ligands of CD28; extrinsically, it transmits inhibitory signals in reverse to antigen-presenting cells and reduces the expression and availability of the ligands CD80/CD86 (15). CD28 and CTLA-4 constitute a classic regulatory mechanism for T cell activity, which maintains immune homeostasis in the human body under normal conditions (16).

In the TME, Regulatory T cells (Tregs) constitutively express CTLA-4 (17). As key cells that induce immune tolerance and suppress anti-tumor immunity, Tregs exhibit advantages in proliferation and recruitment under the unique hypoxic and acidic TME. Notably, increased Tregs infiltration has been demonstrated to be associated with decreased overall survival rates among patients with ovarian cancer (18). Additionally, Tregs can mediate the transendocytosis of CD80/CD86 via CTLA-4, thereby decreasing the expression of these two ligands on antigen-presenting cells and weakening the strength of costimulation (19). Accordingly, using a specific antibody with high affinity for CTLA-4 as an inhibitor can obstruct the interaction between CTLA-4 and CD80/CD86. This not only mediates Tregs depletion and functional impairment but also attenuates CTLA-4-driven T cell suppression, ultimately enhancing T cell-mediated antitumor immune responses.

PD-1, part of the CD28 family, is mainly found on activated T cells, B cells, and NK cells. Its corresponding ligand, PD-L1, is predominantly present on tumor cells, antigen-presenting cells, and vascular endothelial cells. Within the TME, the binding of tumor cell-derived PD-L1 to T cell-expressed PD-1 triggers PD-1 to recruit Src homology 2 domain-containing protein tyrosine phosphatase 1 (SHP-1) and SHP-2 for intracellular accumulation. This process inhibits the activation of proximal T cell receptor kinases, thereby suppressing downstream signaling pathways (20). Such suppressive signals impair T cell activation and proliferation, reduce cytokine release and alter the TME. As a consequence, PD-L1 expression on the surface of tumor cells becomes further elevated, enabling these cells to evade immune monitoring with greater ease. Therefore, administering PD-1 or PD-L1 inhibitors can disrupt the PD-1/PD-L1 binding, alleviating the inhibitory impact on T cell activation and proliferation, reinstating T cell cytotoxicity and suppressing tumor cell growth.

### 2.2 Current clinical applications and challenges of ICIs

As a major breakthrough in tumor treatment, ICIs have recently shown significant effectiveness in treating both solid tumors and hematological malignancies in recent years. In the field of melanoma treatment, ICIs have completely transformed the therapeutic landscape for patients. In melanoma, for example, ICIs have dramatically reshaped treatment paradigms. Monotherapy with PD-1 inhibitors such as nivolumab and pembrolizumab has led to marked improvements in objective response rates (ORR) and overall survival (OS) in advanced cases (21, 22), with a subset of patients achieving durable long-term survival. As the first approved CTLA-4 inhibitor, the emergence of ipilimumab has also changed the landscape of tumor treatment to a certain extent. Compared with previous traditional chemotherapy and immunotherapy, its use can improve patients’ OS and survival rate (23–25). However, the clinically approved CTLA-4 inhibitors currently available are relatively limited in type and narrow in application scope. Moreover, compared with nivolumab, ipilimumab exhibits limited efficacy and safety (26, 27). Furthermore, studies have confirmed that compared with ipilimumab monotherapy for metastatic melanoma, the “O+Y” dual-immune combination therapy (i.e., nivolumab combined with ipilimumab) results in significantly higher ORR and progression-free survival (PFS) (28). Therefore, CTLA-4 inhibitors are mostly used in combination regimens in clinical practice, among which the “O+Y” dual-immune combination therapy is the most widely adopted. In addition, numerous clinical studies on the “O+Y” combination therapy are currently underway. For example, the ongoing clinical trial with ID NCT05310643 aims to evaluate the efficacy of the “O+Y” treatment regimen in patients with metastatic colorectal cancer who are resistant to anti-PD-1 monotherapy. This indicates that the “O+Y” combination therapy holds considerable potential in clinical applications.

Nevertheless, the clinical application of ICIs still faces challenges. One study demonstrated that blocking CTLA-4 signaling can increase the expression of costimulatory receptors on tumor-associated dendritic cells. Tregs then undergo local proliferation in response to the elevated costimulatory signals, which offsets the therapeutic benefits for patients (29). Such a mechanism could be one factor contributing to the comparatively low effectiveness of CTLA-4 inhibitors when administered as monotherapies in clinical practice (22).In some phase III clinical trials, ICIs failed to achieve the expected therapeutic outcomes. For example, in the KEYNOTE-240 study, while pembrolizumab monotherapy displayed a tendency toward improved survival in patients with advanced hepatocellular carcinoma, it failed to attain the pre-established statistical significance (30). Additionally, the use of ICIs can lead to irAEs involving multiple systems and organs, such as endocrine side effects (31) and ocular side effects (32). Notably, combination ICIs therapy also significantly increases the incidence of irAEs in patients (28, 33). The study has shown that 95.5%, 82.1%, and 86.2% of patients in the Nivolumab + Ipilimumab combination group, Nivolumab monotherapy group, and Ipilimumab monotherapy group, respectively, experienced any grade of irAEs, among which the incidences of grade 3 or 4 irAEs were 55%, 16.3%, and 27.3% respectively (33).

Therefore, improving the effectiveness of ICIs and minimizing irAEs represent a pressing challenge in current tumor immunotherapy. As a unique therapeutic approach, TCM holds significant potential and may offer effective complementary strategies to address this challenge.

## 3 TCM assists ICIs therapy by enhancing drug delivery

TME is central to the initiation, progression, and metastasis of tumors, functioning as a complex ecosystem that includes tumor cells, immune cells, stromal cells, and the extracellular matrix (ECM). Within this environment, the functional activity of immune cells is regulated by multiple factors, which often induce immunosuppression and enable tumor cells to evade the body’s immune surveillance. Solid tumors possess unique structural features, including abnormally proliferative vascular systems, dense ECM, high cell density, and a hypoxic acidic microenvironment. These characteristics impede effective drug delivery to the tumor interior (4), which may be a critical factor limiting the efficacy of ICIs. Therefore, modifying the unique TME of solid tumors stands as an effective strategy to improve drug delivery and enhance the performance of ICIs.

### 3.1 Promotion of vascular normalization

Promoting vascular normalization represents an emerging pharmacological target for improving drug delivery and enhancing antitumor immune responses (34).As bioactive substances extracted from Chinese herbal medicines, TCM monomers exhibit distinct advantages over synthetic compounds, including high efficacy, low toxicity, and multi-targeting capabilities. Consequently, research on TCM monomers has become a primary focus in investigating the mechanisms underlying TCM-mediated disease treatment. Panaxsaponin, a monomer extracted from traditional Chinese medicine (TCM), has been demonstrated in the study to downregulate the expression of the tumor vascular-related gene EphA, promote the normalization of tumor blood vessels, and inhibit the growth and proliferation of breast cancer tissue (35). The research team led by Professors Yin Lu and Yang Zhao from Nanjing University of TCM found that salvianic acid A improves the structural and functional abnormalities of vascular endothelial cells by regulating pyruvate kinase and its downstream signaling axis, thereby promoting vascular normalization and enhancing the delivery of the chemotherapeutic drug doxorubicin within tumor tissues (36). In combination with ICIs, both tanshinone IIA and salvianic acid B have been shown to promote vascular normalization. Specifically, tanshinone IIA, a lipophilic diterpenoid, disrupts EphA2 signaling, boosting ICI perfusion (37), while salvianic acid B could improve the delivery of cisplatin and exhibit synergistic effects with PD-L1 inhibitors in inhibiting tumor growth (38). In addition, numerous studies have demonstrated that flavonoid and alkaloid TCM monomers can reduce tumor angiogenesis. For instance, baicalin, baicalein, and berberine can effectively inhibit tumor angiogenesis and tumor cell growth by suppressing the VEGF, ERK, and PI3K/Akt signaling pathways (39, 40). Although they have not been explicitly shown to promote vascular normalization or drug delivery, since the abnormal structure of tumor blood vessels is largely attributed to abnormal vascular proliferation, inhibiting tumor angiogenesis can improve the function of tumor blood vessels to a certain extent (41). This may also serve as a new research direction for subsequent studies on vascular normalization and combined drug applications.

Of course, TCM treatment does not rely solely on TCM monomers. In clinical practice, the active components of TCM formulas are diverse, and interactions among different Chinese herbal medicines are complex. Therefore, the guidance provided by TCM monomer research for clinical formulas has certain limitations. An increasing number of studies are now investigating the effectiveness and mechanisms of TCM compound formulas. A study shows that the combination of Astragali (a Qi-invigorating herb) and Curcumae (a blood-activating herb) can reduce glycolysis in colorectal cancer cells and tumor-associated endothelial cells by inhibiting HIF-α, restore endothelial cell integrity, thereby promoting vascular normalization and reducing colorectal cancer metastasis (42). Although this study has not been clearly confirmed whether the combination of Astragali and Curcumae exerts a synergistic improvement effect on the therapeutic efficacy of ICIs or chemotherapeutic drugs, the distinct pro-angiogenic normalization effect exhibited by this combination has provided an important theoretical basis for TCM compound formulas to improve the TME of solid tumors. Moreover, another study indicates that Taohong Siwu Decoction can significantly improve the tumor vascular architecture in breast cancer-bearing mice and enhance the anti-tumor efficacy of the chemotherapeutic drug doxorubicin (43).

In addition, peritumoral electroacupuncture treatment can downregulate GOL1 expression in endothelial cells, inhibit the pyruvate-methylglyoxal-glycolysis pathway in endothelial cells, thereby regulating angiogenic factors and promoting vascular normalization. When combined with the chemotherapeutic drug paclitaxel on the third day after electroacupuncture treatment, it can significantly boost the anti-tumor efficacy of paclitaxel (44). Moxibustion can promote vascular normalization by downregulating HIF-1α and VEGF, and can also significantly boost the anti-tumor efficacy of cisplatin (45).

Over the past decade, various studies have examined the impact of TCM-based therapies on tumor vascular normalization (details is depicted in **Table 1**). These studies fully demonstrate that TCM can facilitate tumor vascular normalization and inhibit tumor growth through multiple mechanisms. Meanwhile, as an adjuvant therapeutic approach, TCM can also augment the anti-tumor effectiveness of chemotherapeutics and ICIs.

**Table 1:** Studies on TCM monomers, TCM herbs/compounds, and TCM external therapies in the field of promoting tumor vascular normalization in the past decade.

TCM intervention types	Therapeutic drugs/methods	Doses	Tumor	Target/pathway	Molecular mechanism	Therapeutic effect	Reference
TCM monomers (phytochemical classes)	Panax notoginseng saponins (Notoginsenosides)	80 mg/kg; 160 mg/kg	Breast cancer	EphA2	Inhibition of EphA2 downregulates the expression of VEGF, Hif-1α, MMP-9, and Smad2/3.	Promotes vascular normalization	(35)
	Salvianic acid A (Phenolic acids)	40 mg/kg	Melanoma and lung cancer	PKM2/β-Catenin/Claudin-5	By binding to PKM2, it activates the β-Catenin/Claudin-5 signaling axis and blocks endothelial cell glycolysis.	1. Promotes vascular normalization 2. Facilitates the delivery of doxorubicin into tumors and augment the anti-tumor effectiveness	(36)
	Tanshinone IIA (Diterpenoid quinones)	45 mg/kg(low doses); 90 mg/kg(high doses)	Hepatoma	ELTD1	Inhibiting ETLD1 promotes the expression levels of tight junction proteins such as ZO-1, occludin, Claudin 5, and Col IV.	1. Promotes vascular normalization and T-cell infiltration 2. Augments the anti-tumor efficacy of PD-1 inhibitors	(37)
	Salvianolic acid B (Phenolic acids)	10 mg/kg; 40 mg/kg	Breast cancer	Ezh2	Inhibiting the expression of Ezh2 increases the expression of VE-cadherin.	1. Promotes vascular normalization and T-cell infiltration 2. Enhances the delivery of cisplatin and its anti-tumor effect 3. Augments the anti-tumor efficacy of PD-L1 inhibitors	(38)
	Tanshinone II A (Diterpenoid quinones)	5 mg/kg	Hepatoma	\	\	1. Promotes vascular normalization 2. Improves distribution and anti-tumor efficacy of pegylated liposomal doxorubicin	(78)
	Tanshinone IIA (Diterpenoid quinones)	10 mg/kg; 30 mg/kg; 90 mg/kg	Colon cancer	Ang2/Tie2	By inhibiting Ang2 expression in endothelial cells and activating the Tie2 signaling pathway, vascular stability is enhanced.	Promotes vascular normalization	(91)
	Tanshinone IIA (Diterpenoid quinones)	10 mg/kg	Hepatoma	PI3K-AKT	By inhibiting the PI3K-AKT signaling pathway, the expression of HIF-1α and HIF-2α is reduced.	1. Promotes vascular normalization 2. Augments the anti-tumor efficacy of sorafenib	(79)
	Astragali Polysaccharide (Polysaccharides) and Curcumin (Diarylheptanoids)	Astragali Polysaccharide (100 mg/kg); Curcumin (100 mg/kg)	Hepatoma	\	\	Promotes vascular normalization	(92)
	Ginsenoside CK (Triterpenoid saponins)	40 mg/kg	Non-small cell lung cancer	HIF-1α, VEGF, FGF2	Inhibits the expression of HIF-1α, VEGF and FGF2	1. Promotes vascular normalization 2. Augments the sensitivity of tumor tissues to gefitinib	(93)
	6-Gingerol (Gingerols)	100 mg/kg; 200 mg/kg	Hepatoma and breast cancer	VEGFR2	Promotes the formation of the VEGFR2/VE-cadherin/β-catenin/actin complex through targeted binding to VEGFR2.	1. Promotes vascular normalization 2. Augments the anti-tumor efficacy of cisplatin	(94)
	α-mangostin (Xanthones)	10 mg/kg	Pancreatic cancer	TGF-β/Smad	Inhibition of the TGF-β/Smad pathway inactivates CAFs, reduces ECM deposition, and attenuates tumor vascular compression.	1. Promotes vascular normalization 2. Enhances the delivery and anti-tumor effect of triptolide	(52)
TCM herbs/compounds	Astragali and Curcumae	1.95 g/kg (low-dose group); 3.9 g/kg (high-dose group)	Colon cancer	HIF-1α	By inhibiting HIF-1α nuclear translocation to reduce glycolysis in endothelial cells, thereby restoring endothelial cell integrity.	Promotes vascular normalization	(42)
	Taohong Siwu decoction	2.63 g/kg (low dose); 5.26 g/kg (high dose)	Breast cancer	VEGF, HIF-1α	Inhibits the expression of VEGF and HIF-1α	1. Promotes vascular normalization 2. augments the anti-tumor efficacy of doxorubicin 3. Shifts the polarization of macrophages from the M2 phenotype to the M1 phenotype in the TME	(43)
	Xiaoliu Pingyi Recipe	10 ml/kg	Lung adenocarcinoma	HIF-1α, VEGFA, Ang-2	Inhibits the expression of HIF-1α, VEGFA and Ang-2	Promotes vascular normalization	(95)
	Shenmai injection	10 ml/kg	Colon cancer	Histone H3	Downregulation of VEGF, FGF, and PAI-1 expression in tumor tissues by inhibiting histone H3 acetylation.	1. Promotes vascular normalization 2. Enhances the delivery and anti-tumor effect of 5-fluorouracil	(96)
	Dahuang Zhechong pill	The concentration ratio is provided (20g DHZCP is added ultrapure water to 100 mL ultrapure water), but the dosage is not mentioned.	Hepatoma	MK/Itgα	Suppression of the MK/Itgα signaling pathway reduces the expression of VEGF, VEGFR, Ang-2, and Tie2.	Reduces pathological angiogenesis and promotes vascular normalization	(97)
	Qu-Du-San-Jie decoction	Multiplying a factor of 0.026 for mice with reference to body surface area	Vestibular schwannoma	\	\	Promotes vascular normalization	(98)
TCM external therapies	Electroacupuncture	(44): one session, four needles; (99): a 30-minute EA session, four needles	Breast cancer	GLO1	Downregulating GLO1 expression in endothelial cells promotes tumor-associated macrophage polarization toward the M1 phenotype and inhibits the pyruvate-methylglyoxal-glycolytic pathway in endothelial cells.	1. Promotes vascular normalization 2. Augments the anti-tumor efficacy of paclitaxel	(44, 99)
	Moxibustion	Moxibustion performed every other day for a total of 7 sessions	Non-small cell lung cancer	HIF-1α, VEGF	Downregulates the expression levels of HIF-1α and VEGF	1. Promotes vascular normalization 2. Augments the anti-tumor efficacy of cisplatin	(45)

In the Doses column, some studies investigate dose-response relationships, among which the bolded values indicate the doses with better efficacy.

### 3.2 Improvement of the ECM

In the TME, abnormal deposition of ECM is common. A dense and high-stiffness network, which is formed by excessive cross-linking of collagen, fibronectin and other components, could act as an interstitial barrier outside tumor cells, impeding the effective delivery of drugs (46, 47). Additionally, this abnormal ECM deposition can promote the growth and migration of tumor cells (48). Cancer-associated fibroblasts (CAFs), originating from fibroblasts stimulated by tumor cell-secreted growth factors (e.g., TGF-α, TGF-β, FGF-2, PDGF, and EGF), are the main contributors to the extracellular matrix (ECM) in the TME (47, 48). ECM modifications mediated by CAFs not only hinder drug delivery but also directly or indirectly decrease immune cell infiltration within the TME (49).

Therefore, targeting CAFs or related growth factors can reverse the abnormal deposition of the ECM, thereby enhancing both anti-tumor drug delivery and immune cell infiltration (50). This suggests a logical research approach for TCM to improve the anti-tumor effectiveness of ICIs. The study has shown that isochuanliansu, a TCM monomer, can competitively bind to transforming growth factor-β receptor type 1 (TGFβR1), inhibiting TGF-β-mediated downstream signaling pathways and reducing collagen deposition in the TME. This modification markedly improves the anti-tumor effectiveness of PD-L1 inhibitors (51).Another study attempted to design nanoparticle carriers for targeted delivery of the TCM monomer α-mangostin to CAFs. This method enhances drug delivery and anti-tumor effectiveness by targeting the TGF-β/Smad pathway to deactivate CAFs, thereby reducing ECM deposition (52). Similarly, the delivery of quercetin (53) and ginsenoside Rg3 (54) via nanoparticle carriers yields comparable outcomes. In addition, baicalin and baicalein can inhibit the activation of CAFs and reduce the deposition of the ECM (39). This may serve as a research basis for further investigating the targeted delivery of baicalin and baicalein as well as their combination with other drugs.

**Figure 1:**
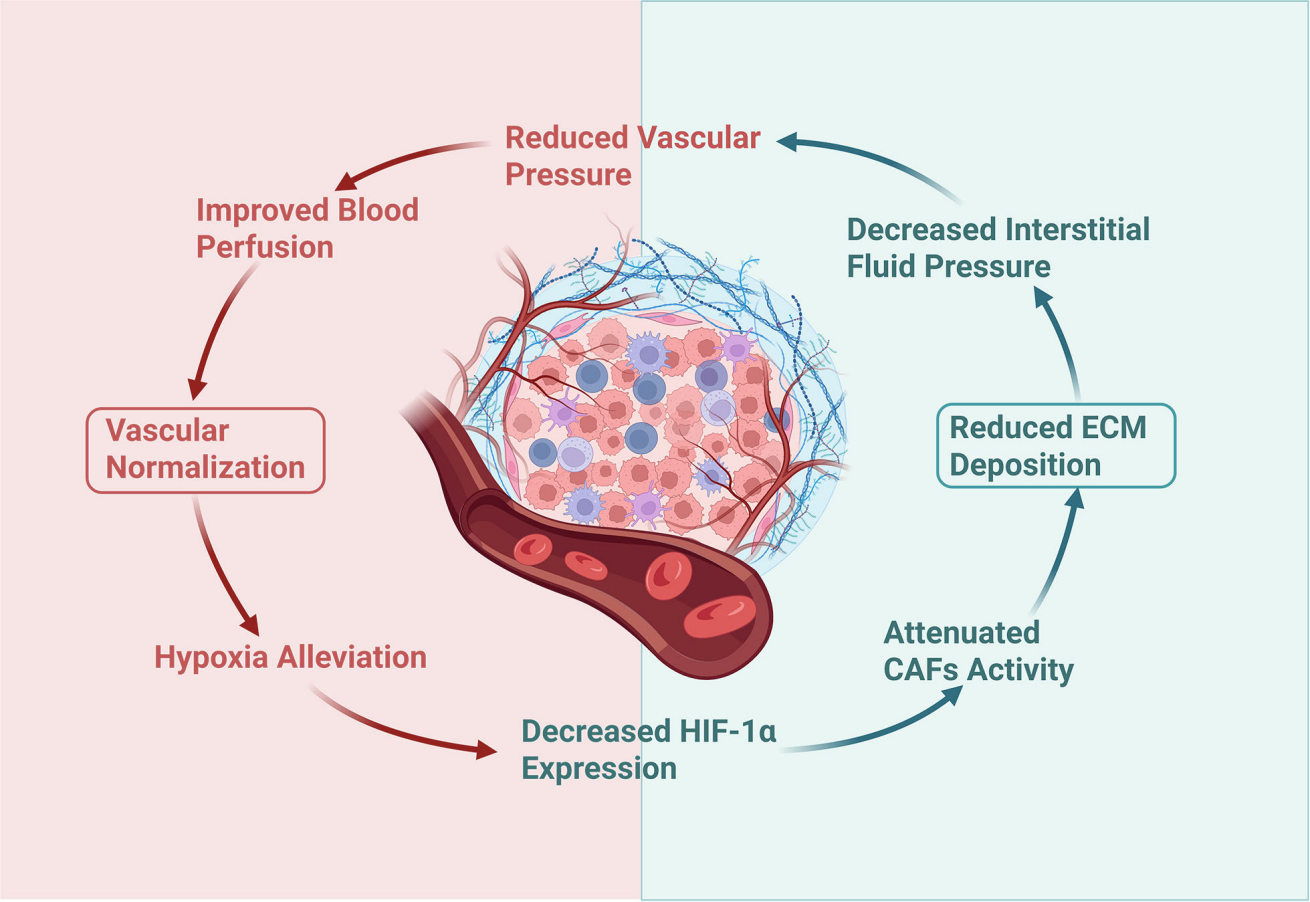
Loop diagram related to the synergistic effect of blood vessels/ECM: Vascular normalization and reduced ECM deposition can form a benign cycle by improving blood perfusion and alleviating tumor tissue hypoxia. Created in BioRender. Liu, Z (2025). https://BioRender.com/2kbd6yn.

However, the ECM impedes drug delivery not merely by forming a interstitial barrier, but also potentially through associations with vascular formation and stability (55, 56). Promoting vascular normalization in the TME can alleviate tumor tissue hypoxia, and inhibit the expression of HIF-1α, thereby suppressing HIF-1α-driven activation of CAFs and further reducing abnormal ECM deposition (57, 58). Meanwhile, the reduced ECM deposition alleviates its compression on tumor blood vessels, reduces vascular pressure, improves blood perfusion, and promotes vascular normalization (52). Thus, a self-reinforcing cycle of “vascular normalization → alleviated hypoxia → reduced ECM deposition → decreased vascular pressure → vascular normalization” is formed (**Figure 1**). Therefore, promoting vascular normalization and improving the ECM are two mutually reinforcing approaches to enhance drug delivery, and TCM has demonstrated remarkable efficacy in both aspects.

## 4 TCM assists ICIs by promoting infiltration and activation of immune cells

The core function of ICIs is to reverse the exhaustion and suppression of immune cells induced by tumor cells. Consequently, for ICIs to exert their therapeutic effects, an adequate population of functional immune cells must be present within the TME (59). Additionally, numerous studies have demonstrated that promoting immune cell infiltration and activation can significantly enhance the ICIs’ efficacy (60–62).

TCM also exhibits remarkable efficacy in promoting immune cell infiltration and activation. As mentioned earlier, the promotion of tumor vascular normalization and improvement of the ECM induced by TCM can not only facilitate drug delivery but also enhance immune cell infiltration (37, 38, 43, 51). In addition, TCM can directly stimulate immune cell activation and increase immune cell infiltration by promoting chemokine secretion or reducing T cell exhaustion. Studies have shown that Liujunzi Decoction can significantly decrease CD3^+^PD-1^+^T cell proportions in tumor tissues, reverse the exhausted phenotype of T cells, and enhances IFN-γ expression in the TME. These effects further drive the activation of CD3^+^CD8^+^ T cells and strengthen their capacity to infiltrate tumor tissues (63). Sijunzi Decoction can inhibit tumor PD-L1 expression by downregulating the TLR4/MyD88/NF-κB signaling pathway in tumors while upregulating IL-2 expression, thereby enhancing the T cells’ immune response (64). Other research indicates that Qizhen Decoction promotes dendritic cell maturation through the gut microbiota, activates the IL-12/JAK2/STAT4 pathway, further induces T cell polarization and activation, and significantly improves the efficacy of PD-1 inhibitors in treating colorectal cancer (65). Shenling Baizhu Decoction drives macrophage polarization toward the M1 phenotype and reduces regulatory Tregs production, thereby exerting a synergistic effect with PD-1 inhibitors (66). Astragalus polysaccharide, a TCM monomer, enhances dendritic cell maturation by upregulating MHC and co-stimulatory molecules, thereby improving antigen presentation and T cell activation (67). Ginsenoside Rh2 can increase the chemokine CXCL10 expression to facilitate CD8^+^ T cell infiltration into tumors and significantly improve PD-L1 inhibitor efficacy (68). Beyond pharmaceutical interventions, the latest research has demonstrated that electroacupuncture can effectively improve the immune status in the TME by activating the STING pathway, increase the infiltration of CD8^+^ T cells and NK cells in the TME, and thus enhance the efficacy of PD-1 inhibitors (69).

## 5 Conclusion

### 5.1 Summary and outlook of TCM-assisted ICIs

**Figure 2:**
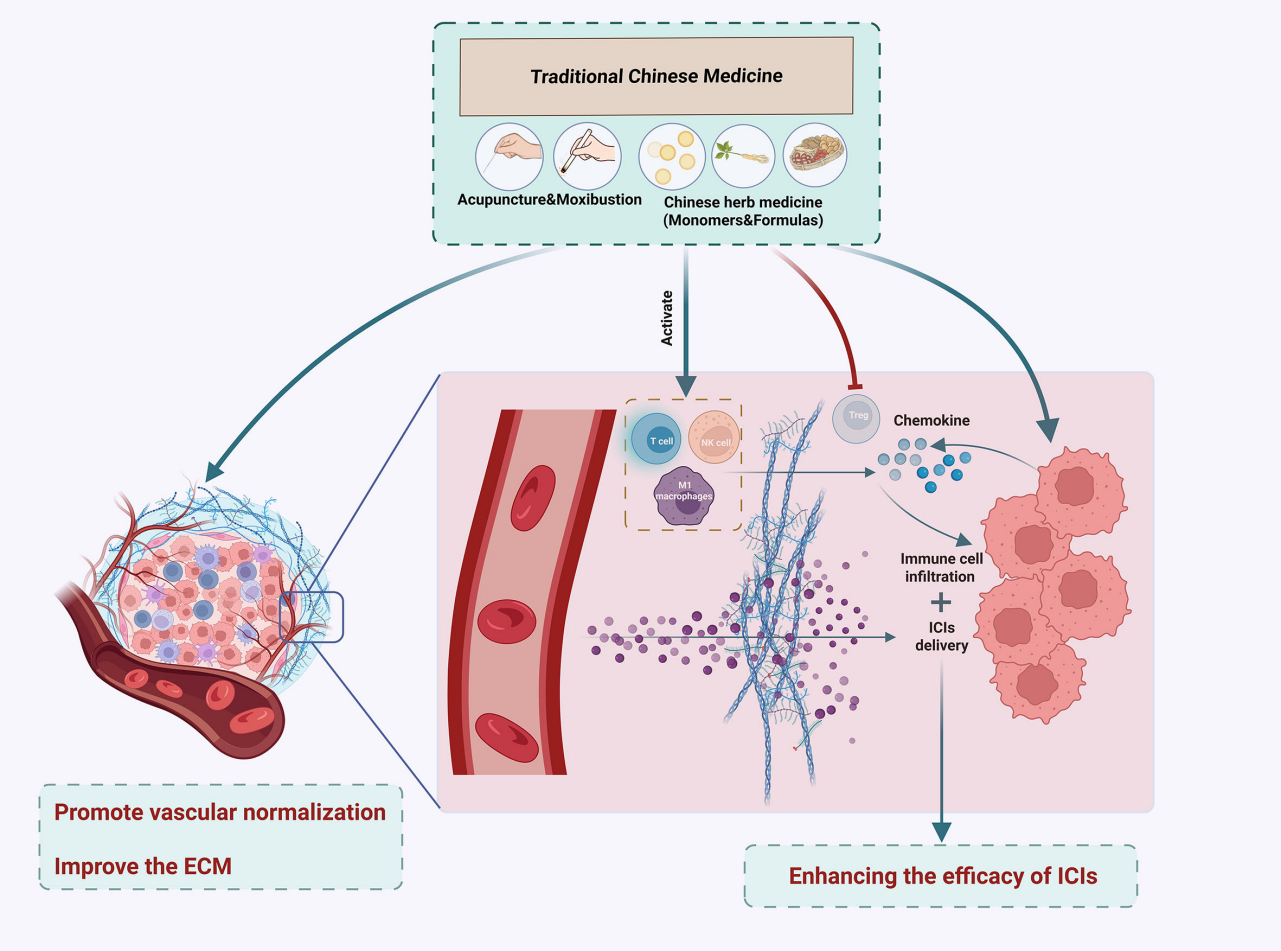
Mechanism diagram of TCM enhancing the efficacy of ICIs: TCM can promote the delivery of ICIs and the infiltration of immune cells by facilitating vascular normalization and improving the extracellular matrix. In addition, TCM can activate immune cells, inhibit Tregs, and stimulate tumor cells to secrete chemokines, thereby promoting the recovery of immune cell function and their infiltration into tumor tissues. Created in BioRender. Liu, Z (2025). https://BioRender.com/xlqanon.

Existing studies have fully confirmed that TCM serves a definite function in assisting ICIs for solid tumors. It can improve the efficiency of targeted drug delivery and enhance the infiltration capacity and activation level of immune cells within the TME (**Figure 2**), thereby providing crucial support for the anti-tumor efficacy of ICIs.

Of greater clinical value, combination therapy of ICIs with TCM can reduce the toxicity grade and the incidence of various irAEs induced by ICIs (12). For example, Gegen Qinlian Tablets can attenuate the incidence of irAEs in patients with advanced NSCLC receiving ICIs treatment (70). Moreover, as the most common irAE during ICIs treatment (71), skin-related adverse events can have their symptoms alleviated and incidence reduced by TCM through the method of clearing heat and resolving dampness (70, 72). Combined with TCM’s unique advantages, such as high ease of use and controllable treatment costs, it further confirms that the strategy of TCM-assisted ICIs therapy for solid tumors holds promising prospects and feasibility in clinical translation and popularization. However, due to the specific differences among different solid tumors, treatment strategies for solid tumors cannot be generalized. For example, vascular normalization strategies are more suitable for hypoxic hepatocellular carcinoma (73), whereas for pancreatic cancer with a dense ECM, the treatment focus should be on the ECM (74). Therefore, many current studies have attempted to construct nanocarriers to load TCM drugs for enhancing their delivery efficiency and targeting ability (52–54), which will provide more precise treatment for solid tumors with different characteristics. In addition, biomarkers for patients sensitive to the TCM-ICIs combinations can be developed in the future, which will facilitate the screening of patients most likely to benefit from the TCM-ICIs combinations during future clinical translation to achieve precision medicine.

Furthermore, most current studies on TCM-assisted ICIs therapy for solid tumors focus on the PD-1/PD-L1 pathway, with relatively insufficient exploration of emerging targets such as TIGIT, LAG-3, and Clever-1.The role of these targets in tumor immunotherapy cannot be ignored (60, 75–77). Future studies can use TCM libraries to identify compounds that can alter the expression of TIGIT, LAG-3, and Clever-1.

### 5.2 Critical appraisal on the mechanisms of TCM-assisted ICIs

In addition to investigating the mechanisms by which TCM exerts positive adjuvant effects on ICIs therapy for tumors, we must also face up to the current limitations in preclinical studies, such as the lack of standardization and heterogeneity of results. For instance, among numerous studies on Tanshinone IIA in treating hepatoma model mice, differences in cell lines, tumor modeling methods, and drug dosages have hindered the establishment of a standardized treatment protocol (37, 78, 79). Moreover, the synergistic effects of some TCMs with ICIs have shown significant inconsistencies. For example, one study demonstrated that Astragalus injection can antagonize the efficacy of anti-PD-1 against melanoma through inhibiting the JAK/STAT signaling pathway and down-regulating MHC-II expression (80), which is in stark contrast to another study showing that Astragalus membranaceus polysaccharides enhance ICI efficacy by increasing intratumoral CD8+ T cells infiltration and reducing the proportion of Tregs (81). Furthermore, certain TCMs exhibit immunosuppressive properties, such as extracts of Tripterygium wilfordii Hook (82). Therefore, greater attention should be paid to the application of these TCMs during studies and clinical treatment.

The aforementioned issues all result in limitations in the clinical translation of TCM-assisted ICIs therapy. I have retrieved completed or ongoing clinical trials of TCM (including TCM formulas or acupuncture) combined with ICIs for tumor treatment (**Table 2**), and found that clinical evidence is severely insufficient. Moreover, most of the current evidence confirming that TCM can effectively enhance ICIs efficacy is derived from preclinical studies. Therefore, to further advance clinical translation, large-sample, rigorous randomized controlled clinical trials are indispensable.

**Table 2:** Completed or ongoing clinical trials investigating TCM (including Chinese herbal formulas or acupuncture) combined with ICIs for anti-tumor therapy.

Trial ID	Cancer type	TCM intervention	ICI used	Primary outcomes	Key results	Reference/link
NCT05735028	NSCLC	Centipeda minima	PD-1/PD-L1 inhibitor	Progression-free survival, the temperature, blood pressure, complete blood count, adverse event and severe adverse event	\	https://clinicaltrials.gov/study/NCT05735028
NCT07034326	NSCLC	Electroacupuncture and Zilongjin tablet	Including but not limited to pembrolizumab and atezolizumab	Disease-free survival	\	https://clinicaltrials.gov/study/NCT07034326
NCT06992024	Gastric Cancer	Electroacupuncture	PD-1 inhibitor + Paclitaxel Protein-bound	Objective response rate	\	https://clinicaltrials.gov/study/NCT06992024
NCT05834413	NSCLC	Phase1:HeWeiYangXueFang Phase 2:FeiPingFang	ICIs + chemotherapy	Disease-free survival	\	https://clinicaltrials.gov/study/NCT05834413
NCT07239661	NSCLC	Electroacupuncture	PD-1 inhibitor	Progression-free survival	\	https://clinicaltrials.gov/study/NCT07239661
NCT07086300	NSCLC	Electroacupuncture	PD-1 inhibitor	Progression-free survival	\	https://clinicaltrials.gov/study/NCT07086300
NCT06461338	NSCLC	Acupuncture	ICIs + chemotherapy	Progression-free survival	\	https://clinicaltrials.gov/study/NCT06461338
NCT06249854	NSCLC	Bojungikki-tang (Buzhong Yiqi Decoction)	Pembrolizumab	Progression-free survival	BJIKT may enhance immune response and potentially improve clinical outcomes in patients with NSCLC receiving immune checkpoint inhibitor therapy.	(100)
NCT07076836	NSCLC	Electroacupuncture (ST36)	PD-1 inhibitor	Neoadjuvant immunotherapy conversion rate	\	https://clinicaltrials.gov/study/NCT07076836
ChiCTR2200062607	NSCLC	Gegen Qinlian Tablets	ICIs + chemotherapy	Incidence and severity of irAEs	GQT significantly reduced the incidence of irAEs and prolonged the median onset time of irAEs in patients with advanced NSCLC receiving ICI therapy.	(70)
ChiCTR2300069345	NSCLC	Fuzheng Kang’ai prescription	PD-1/PD-L1 inhibitor + chemotherapy	Incidence of adverse reactions	\	https://www.chictr.org.cn/showproj.html?proj=190960
ChiCTR2300068896	Gastric Cancer	Astragalus	PD-1 inhibitor + chemotherapy	Objective response rate	\	https://www.chictr.org.cn/showproj.html?proj=189330
ITMCTR2025000614	Non-small cell lung cancer	Lumaiyifei Formula	ICIs + chemotherapy	Disease control rate	\	https://itmctr.ccebtcm.org.cn/mgt/project/view/-1168191489277532349

### 5.3 Pharmacokinetic interactions and safety considerations

When TCMs is used in combination with other anti-cancer drugs, pharmacokinetic research is also indispensable. TCM components can influence the efficacy of combined drugs by regulating metabolic enzymes, for example, the main components in ginseng can significantly upregulate CYP3A4 (83–85), which, as a key catalyst in the metabolism of many drugs, catalyzes the activation and inactivation of various anti-cancer drugs. TCMs standardization and quality control are crucial for clinical research and application. Non-standardized TCMs may exhibit significant fluctuations in active ingredients due to differences in cultivation and processing (86, 87), which could interfere with the research conclusions on pharmacokinetic interactions. We can achieve standardized preparations through fingerprints technology to ensure the efficacy and safety of drugs (88). Meanwhile, we cannot ignore the issue that certain TCMs may induce excessive immune activation, leading to autoimmune liver injury (89, 90), which could potentially affect the metabolism of combined drugs and exacerbate toxic reactions.
